# Establishment and validation of a prediction nomogram for heart failure risk in patients with acute myocardial infarction during hospitalization

**DOI:** 10.1186/s12872-023-03665-2

**Published:** 2023-12-18

**Authors:** Shengyue Chen, Xinling Pan, Jiahang Mo, Bin Wang

**Affiliations:** 1 Author affiliations Dalian Medical University, Dalian, Liaoning China; 2 Author affiliations Department of Biomedical Sciences Laboratory, Wenzhou Medical University Affiliated Dongyang Hospital, Dongyang, Zhejiang, China; 3 Author affiliations Obstetrics and Gynecology Hospital, Fudan University, Shanghai, China; 4 Author affiliations Department of Hepatobiliary Surgery, Wenzhou Medical University Affiliated Dongyang Hospital, Dongyang, Zhejiang China

**Keywords:** Acute myocardial infarction, Nomogram, Heart failure, Prediction model, Influencing factors

## Abstract

**Background:**

Acute myocardial infarction (AMI) with consequent heart failure is one of the leading causes of death in humans. The aim of this study was to develop a prediction model to identify heart failure risk in patients with AMI during hospitalization.

**Methods:**

The data on hospitalized patients with AMI were retrospectively collected and divided randomly into modeling and validation groups at a ratio of 7:3. In the modeling group, the independent risk factors for heart failure during hospitalization were obtained to establish a logistic prediction model, and a nomogram was constructed. The receiver operating characteristic (ROC) curve, calibration curve, and decision curve analysis (DCA) were used to evaluate the predictive performance and clinical value. Machine learning models with stacking method were also constructed and compared to logistic model.

**Results:**

A total of 1875 patients with AMI were enrolled in this study, with a heart failure rate of 5.1% during hospitalization. The independent risk factors for heart failure were age, heart rate, systolic blood pressure, troponin T, left ventricular ejection fraction and pro-brain natriuretic peptide levels. The area under the curve (AUC) of the model in modeling group and validation group were 0.829 and 0.846, respectively. The calibration curve showed high prediction accuracy and the DCA curve showed good clinical value. The AUC value of the ensemble model by the stacking method in the validation group were 0.821, comparable to logistic prediction model.

**Conclusions:**

This model, combining laboratory and clinical factors, has good efficacy in predicting heart failure during hospitalization in AMI patients.

**Supplementary Information:**

The online version contains supplementary material available at 10.1186/s12872-023-03665-2.

## Introduction

As one of the most common causes of death, acute myocardial infarction (AMI) is a serious disease [1, 2]. AMI leads to 6.4 million deaths per year in the USA and Europe, and it accounts for more than a third of deaths in other developed countries [3, 4]. According to the China Cardiovascular Health and Disease Report 2021, AMI rose from 23.2/100,000 in 2003 to 62.33/100,000 in 2008 for urban residents in China. AMI results bring heavy burdens to family and society due to its high cost in the treatment. Moreover, baseline diseases such as diabetes would increase the mortality of patients with AMI, which could be explained that diabetes might accelerate the process of atherosclerosis [5, 6].

Heart failure is a common complication of AMI. It is characterized by severe systolic and/or diastolic function impairment of the heart [7, 8]. The progression from AMI to heart failure involves myocardial shock, remodeling, and chronic neuroendocrine system activation [9]. AMI impairs the ventricle function through wall thinning of the infarct zone, ventricular dilatation, and ultimately compensatory hypertrophy and fibrosis [10]. The larger the infarct size leads to heavier burden on the remaining viable myocardium, consequently contributing to higher probability of heart failure [11].

Previous studies have reported that being complicated with heart failure increased the mortality rate of 10 times higher for patients with acute coronary syndrome [12]. It is thus particularly important to predict the risk of heart failure in patients with AMI. Recent studies have shown that carbohydrate antigen 125, brain derived neurotrophic factor, serum soluble growth stimulation expressed gene 2, and interleukin-33 are effective predictors of heart failure in patients with AMI [12, 13]. However, these indicators are rarely measured in clinical trials, which largely limits their clinical practice. In another way, the clinical indexes available from routine laboratory tests during admission would be convenient for evaluating the risk of heart failure, but previous models hardly test the linear relationship between involved variables and terminal event or any interaction among the involved variables. Furthermore, the small sample size or the recruitment of certain subpopulation limits the reliability of the established prediction models to general patients with AMI [14]. In this study, the risk factors related to heart failure in patients with AMI after admission were combined to develop a logistic prediction model and a nomogram was established to evaluate how much the involved indexes contribute to heart failure. This model will help clinicians to predict the possibility of heart failure in patients with AMI during hospitalization and make personalized measures in their therapy process.

## Material and methods

### Patient inclusion and exclusion

Patients with AMI admitted to Dongyang People’s Hospital from January 2010 to September 2022 were included. Patients with AMI were diagnosed upon getting two of three points: clinical manifestations including chest pain and chest distress; abnormal electrocardiogram indicating for ST segment elevation and complete left bundle branch block; troponin or creatine kinase isoenzyme levels higher than 2 times the upper limitation range [15, 16]. Patients who met one of the following exclusion criteria were removed: a previous history of heart failure; existence of heart failure within 24 h after admission, indicated from by clinical manifestation and laboratory examination; presence of cancer, mental illness, or other serious complications; missing data; no myocardial infarction confirmation by emergency percutaneous coronary intervention (PCI). The terminal event was heart failure during hospitalization, and its diagnosis was based on guidelines from the European Society of Cardiology [17]. Heart failure was diagnosed during hospitalization based on the presence of risk factors, corresponding symptoms or signs, abnormal electrocardiogram, BNP ≥ 35 pg/mL and abnormal findings of echocardiography [17]. This study was approved by the Ethics Committee of Dongyang People’s Hospital. Written informed consent for participation was not required for this study due to its retrospective design, and the study was undertaken in accordance with national legislation and institutional requirements.

### Observation indicator collection from a clinical record information mining database

Patient information was extracted retrospectively from a clinical record information mining database (supported Le 9 Co., Ltd.) and included sex, age, history of non-ST segment elevation myocardial infarction (NSTEMI). Laboratory examination results within 24 h after admission were collected, including troponin T, creatine kinase isoenzyme (CKMB), D-dimer, B-type pro-brain natriuretic peptide, aspartate aminotransferase, high-sensitivity C-reactive protein (CRP), creatinine, white blood cell (WBC) count, platelets, hematocrit. Physical examination was performed to get systolic blood pressure (SBP), left ventricular ejection fraction (LVEF) and heart rate. The treatment intervenes for AMI such as emergency PCI (PCI treatment performed within 12 hours after the onset of the disease is defined as emergency PCI treatment) was also included in this study. Each index unit was converted to the current international unit.

### Variable screening for inclusion in the established prediction model

RStudio 4.4.1 software and IBM SPSS 25.0 software were used to process the data. After collecting the patient data, the variables with missing data more than 25% were removed. The remaining missing variables were interpolated by multiple imputation function as previously described [18]. Count data have been expressed as numbers (percentages), and measurement data have been represented as medians (interquartile ranges). Following univariate analysis (“twogrps” function in CBCgrps package), the boxTidwell function was used to analyze whether there was a linear relationship between significant variables in the univariate analysis and logitp. A multicollinearity test was then performed to test for the presence of multicollinearity between the involved variables, and variance inflation factors (VIFs) of less than 10 indicated no collinearity [19]. Finally, logistic and bidirectional stepwise regression analyses were used to obtain the independent risk factors, and a nomogram was constructed. The area under the curve (AUC) of the receiver operating characteristic (ROC) curve was used to evaluate the discrimination ability of the established model. At the optimal cutoff value, the prediction accuracy, positive prediction value (PPV) and negative predictive value (NPV) were calculated. Calibration evaluation was used to determine whether the predicted probability was consistent with the observed probability. In addition, a decision curve analysis (DCA) was conducted to evaluate the clinical benefit of the nomogram. A *P* value less than 0.05 was considered significant. Also, the model based on stacking method was established in the validation group, which combined the advantages of SVM, C5.0 and Xgboost methods as described previously [20, 21]. The importance of involved variables in the model was evaluated by SHAP summary plots. Finally, the differences among the discrimination powers of established models were tested by using Delong test based on the AUCs.

## Results

### Baseline characteristics of subjects

A total of 1875 patients were enrolled in this study, including 1313 patients in the modeling group, and 562 patients in the validation group. The global heart failure rate was 5.1% (97/1875). The clinical characteristics were comparable between the modeling and validation groups (Table 1).

**Table 1:** Baseline characteristics comparison on patients in the modeling group and the validation group^a^

Variables	Validation group (*n* = 562)	Modeling group (*n* = 1313)	*p*
gender, n (%)			0.315
female	172 (31)	370 (28)	
male	390 (69)	943 (72)	
age, (years)	67 (53, 79)	68 (54, 79)	0.13
NSTEMI			0.087
no	444 (79)	1083 (82)	
yes	118 (21)	230 (18)	
heart failure	27(4.8)	70(5.3)	0.637
systolic blood pressure			0.6
90-140 mmHg	328 (58)	759 (58)	
< 90 mmHg	14 (2)	24 (2)	
> 140 mmHg	220 (39)	530 (40)	
heart rate, (beats/minute)	78.5 (69, 90)	79 (68, 90)	0.702
PCI			0.869
no	256 (46)	591 (45)	
yes	306 (54)	722 (55)	
troponin T, (ng/mL)	0.23 (0.06, 0.85)	0.21 (0.05, 0.86)	0.512
creatine kinase isoenzyme			0.848
< 25 ng/ml	252 (45)	603 (46)	
25-150 ng/ml	264 (47)	611 (47)	
> 150 ng/ml	46 (8)	99 (8)	
D-Dimer, mg/L			0.939
< 2 mg/L	488 (87)	1140 (87)	
2–5 mg/L	53 (9)	120 (9)	
> 5 mg/L	21 (4)	53 (4)	
pro-brain natriuretic peptide			0.721
< 500 ng/L	249 (44)	593 (45)	
500-15,000 ng/L	283 (50)	640 (49)	
> 15,000 ng/L	30 (5)	80 (6)	
aspartate aminotransferase, (U/L)	234 (192, 342.75)	237 (192, 332)	0.801
creatinine			0.915
< 100 μmol/L	453 (81)	1056 (80)	
100-200 μmol/L	94 (17)	216 (16)	
201-400 μmol/L	12 (2)	29 (2)	
> 400 μmol/L	3 (1)	12 (1)	
C-reactive protein			0.06
< 30 mg/L	481 (86)	1071 (82)	
30-80 mg/L	51 (9)	169 (13)	
> 80 mg/L	30 (5)	73 (6)	
hematocrit			0.827
0.3–0.45 L/L	399 (71)	914 (70)	
< 0.3 L/L	29 (5)	69 (5)	
> 0.45 L/L	134 (24)	330 (25)	
white blood cell (× 10^9^/L)	9.52 (7.45, 11.93)	9.42 (7.1, 11.93)	0.402
platelets (×10^9^/L)	211.5 (171.25, 254)	209 (171, 255)	0.464
left ventricular ejection fraction, (%)	57 (50, 63)	57 (48, 63)	0.719

*Abbreviation*: *NSTEMI* Non-ST segment elevation myocardial infarction

### Variable screening by univariate and multivariate analyses

Univariate analysis showed there were 12 indicators significantly related to heart failure in the modeling group (Table 2) (*P* <  0.05). There were linear relationships between 12 variables and logitp (Supplementary Table 1), but no multiple-collinearity was found between the variables (Supplementary Table 2). Logistic and stepwise regression analyses showed that six variables (troponin T, age, heart rate, SBP, LVEF and pro-brain natriuretic peptide) were independent risk factors of heart failure (Table 3) (*P* <  0.05). Consequently, a logistic prediction model was constructed.

**Table 2:** Univariate analysis of enrolled variables between heart failure and control group^a^

Variables	Control (*n* = 1247)	Heart failure (*n* = 66)	*p*
gender, n (%)			0.169
female	346 (28)	24 (36)	
male	901 (72)	42 (64)	
age, (years)	67 (54, 78)	80 (74, 84)	< 0.001
NSTEMI			0.984
no	1028 (82)	55 (83)	
yes	219 (18)	11 (17)	
systolic blood pressure			0.002
90-140 mmHg	725 (58)	34 (52)	
< 90 mmHg	18 (1)	6 (9)	
> 140 mmHg	504 (40)	26 (39)	
heart rate, (beats/minute)	78 (68, 89.5)	93 (80, 105)	< 0.001
PCI			1
no	561 (45)	30 (45)	
yes	686 (55)	36 (55)	
troponin T, (ng/mL)	0.21 (0.05, 0.84)	0.53 (0.14, 2.32)	< 0.001
creatine kinase isoenzyme			0.003
< 25 ng/ml	576 (46)	27 (41)	
25-150 ng/ml	585 (47)	26 (39)	
> 150 ng/ml	86 (7)	13 (20)	
D-Dimer, mg/L			0.053
< 2 mg/L	1089 (87)	51 (77)	
2–5 mg/L	109 (9)	11 (17)	
> 5 mg/L	49 (4)	4 (6)	
pro-brain natriuretic peptide			< 0.001
< 500 ng/L	583 (47)	10 (15)	
500-15,000 ng/L	604 (48)	36 (55)	
> 15,000 ng/L	60 (5)	20 (30)	
aspartate aminotransferase, (U/L)	234 (192, 324.5)	322.5 (237.75, 539.25)	< 0.001
creatinine			< 0.001
< 100 μmol/L	1021 (82)	35 (53)	
100-200 μmol/L	192 (15)	24 (36)	
201-400 μmol/L	25 (2)	4 (6)	
> 400 μmol/L	9 (1)	3 (5)	
C-reactive protein			< 0.001
< 30 mg/L	1029 (83)	42 (64)	
30-80 mg/L	155 (12)	14 (21)	
> 80 mg/L	63 (5)	10 (15)	
hematocrit			0.016
0.3–0.45 L/L	864 (69)	50 (76)	
< 0.3 L/L	62 (5)	7 (11)	
> 0.45 L/L	321 (26)	9 (14)	
white blood cell (×10^9^/L)	9.38 (7.08, 11.86)	10.02 (8.16, 14.46)	0.009
platelets (×10^9^/L)	209 (171, 255)	199.5 (158.25, 244.75)	0.348
left ventricular ejection fraction, (%)	58 (49, 63)	50 (40, 56)	< 0.001

*Abbreviation:* *NSTEMI* Non-ST segment elevation myocardial infarction

**Table 3:** Multivariate logistic regression analysis and Stepwise regression analysis of involved variables in modeling group

Variables	Multivariable logistic regression		Stepwise regression	*P* value
	OR (95%CI)	*P* value	OR (95%CI)	
age, (years)	1.037 (1.012–1.064)	0.005	1.037 (1.014–1.062)	0.002
heart rate, (beats/minute)	1.026 (1.012–1.040)	< 0.001	1.027 (1.014–1.040)	< 0.001
systolic blood pressure				
< 90 mmHg	3.587 (0.945–12.147)	0.047	4.529 (1.197–15.036)	0.017
> 140 mmHg	1.273 (0.705–2.281)	0.418	1.269 (0.716–2.232)	0.41
pro-brain natriuretic peptide				
500–15,000 ng/L	1.565 (0.720–3.630)	0.273	1.62 (0.774–3.649)	0.218
> 15,000 ng/L	2.493 (0.869–7.344)	0.092	3.046 (1.167–8.203)	0.024
troponin T, (ng/mL)	1.067 (0.927–1.213)	0.341	1.133 (0.996–1.232)	0.047
creatine kinase isoenzyme				
25-150 ng/ml	0.692 (0.365–1.299)	0.252	NA	NA
> 150 ng/ml	1.280 (0.447–3.405)	0.632	NA	NA
aspartate aminotransferase, (U/L)	1.000 (1.000–1.001)	0.701	NA	NA
creatinine				
100-200 μmol/L	1.603 (0.843–2.974)	0.140	NA	NA
201-400 μmol/L	1.509 (0.287–5.918)	0.588	NA	NA
> 400 μmol/L	3.258 (0.563–15.519)	0.153	NA	NA
hematocrit				
< 0.3 L/L	1.137 (0.401–2.853)	0.796	NA	NA
> 0.45 L/L	1.059 (0.427–2.402)	0.895	NA	NA
white blood cell (×10^9^/L)	1.023 (0.956–1.090)	0.489	NA	NA
C-reactive protein				
30-80 mg/L	0.844 (0.383–1.756)	0.661	NA	NA
> 80 mg/L	1.053 (0.376–2.658)	0.917	NA	NA
left ventricular ejection fraction, (%)	0.960 (0.936–0.984)	0.001	0.96 (0.937–0.984)	< 0.001

### Establishment of a nomogram from the modeling group

**Fig. 1:**
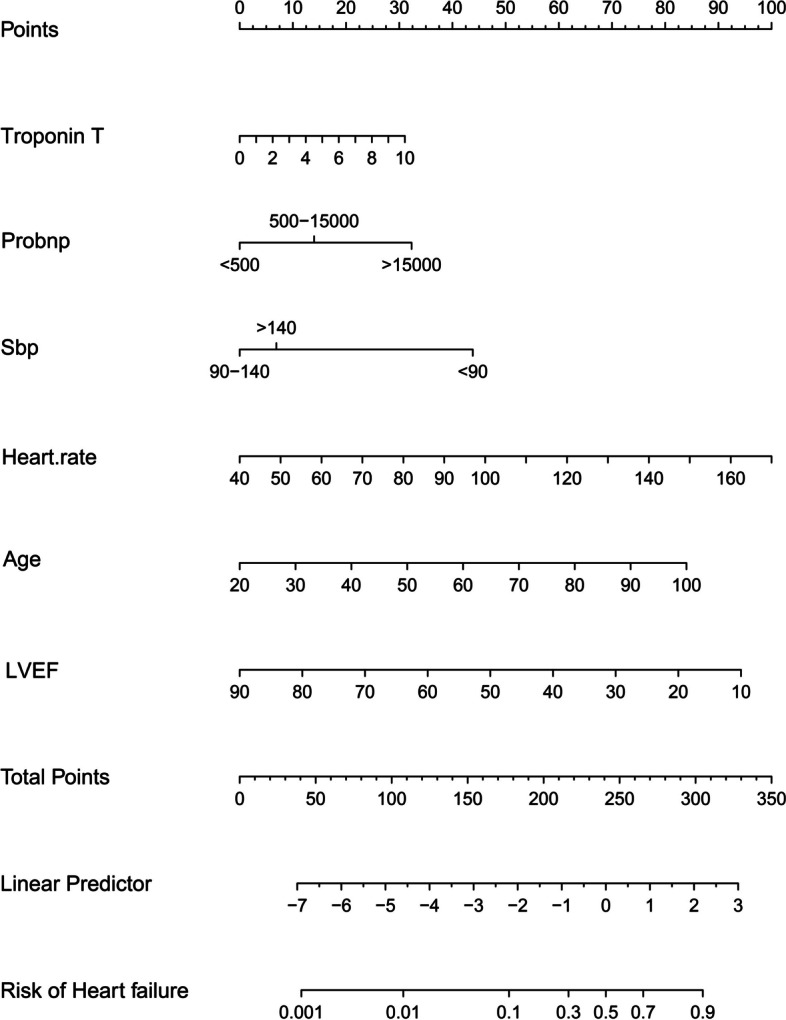
Nomogram graph displaying the contributions of involved variables to the risk of heart failure among patients with AMI during hospitalization

Based on the logistic regression model, a nomogram graph was established by evaluating how much the significant risk factors contributed to the heart failure risk (Fig. 1). Individual points corresponding to the indicated variables were obtained by mapping their detailed results to the upper scoring line; then, the summed score was displayed as the total points. Finally, the risk value of heart failure was obtained by matching the total points to the scoring line at the bottom of the graph.

### Prediction ability of the established model in the modeling validation groups

**Fig. 2:**
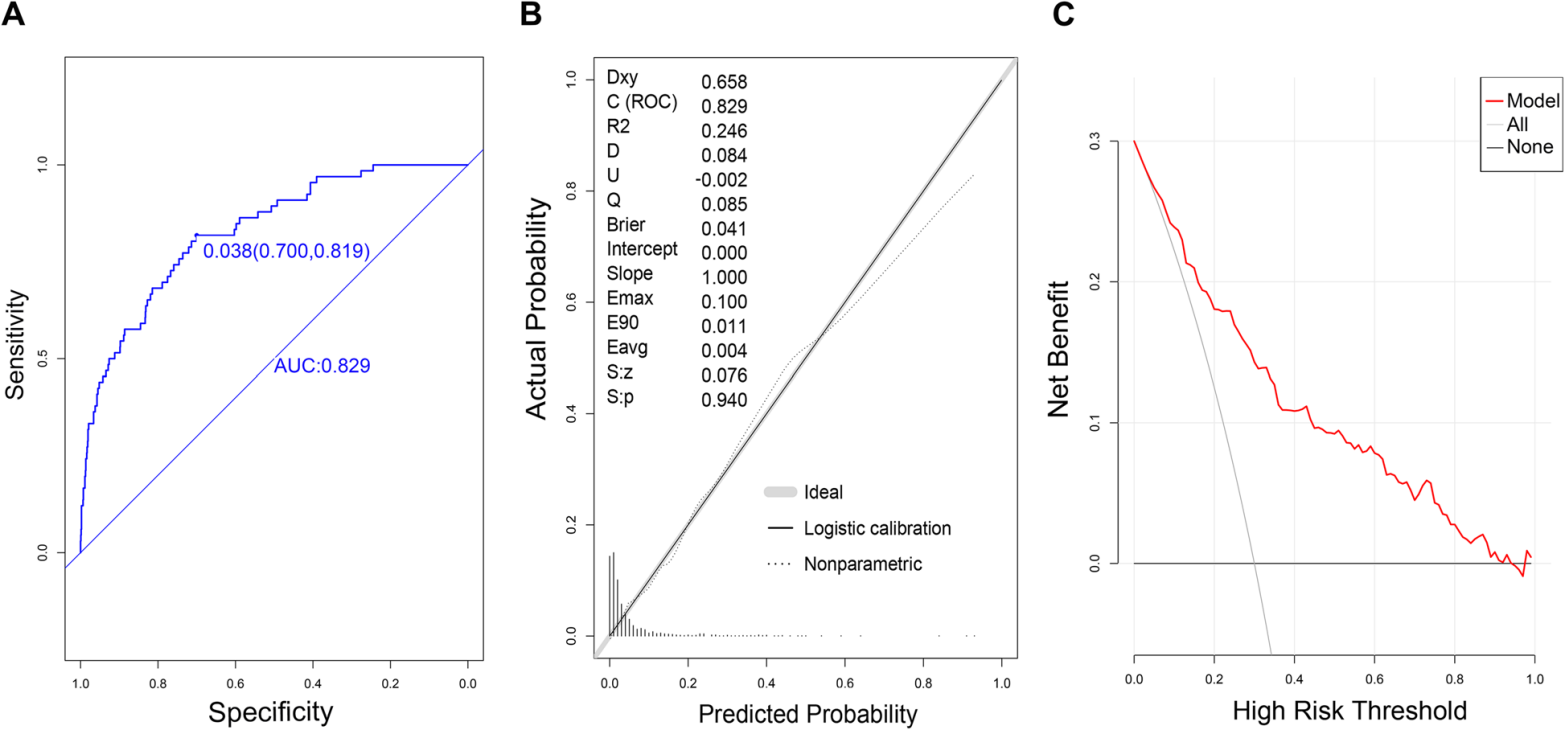
Evaluation of the prediction ability of the established model in the modeling group. **A** Discrimination power indicated by ROC curve; **B** Prediction accuracy indicated by calibration chart; **C** Net clinical benefit indicated by DCA

The AUC value of the model was 0.829 in the modeling group (95% CI: 0.779–0.879), with an optimal cut-off point of 0.038, a sensibility of 70.0%, and a specificity of 81.8%, (Fig. 2A). The prediction accuracy was 0.706 (95% CI:69.6–71.2%), with a PPV of 0.126 (95% CI:9.5–15.8%) and a NPV of 0.986 (95% CI:97.9–99.4%). The *P* value of the calibration curve was 0.940, R2: 0.246, slope: 1.000, Brier: 0.041 (Fig. 2B). DCA curves showed that the model could predict the risk of heart failure with good net clinical benefit (Fig. 2C).

**Fig. 3:**
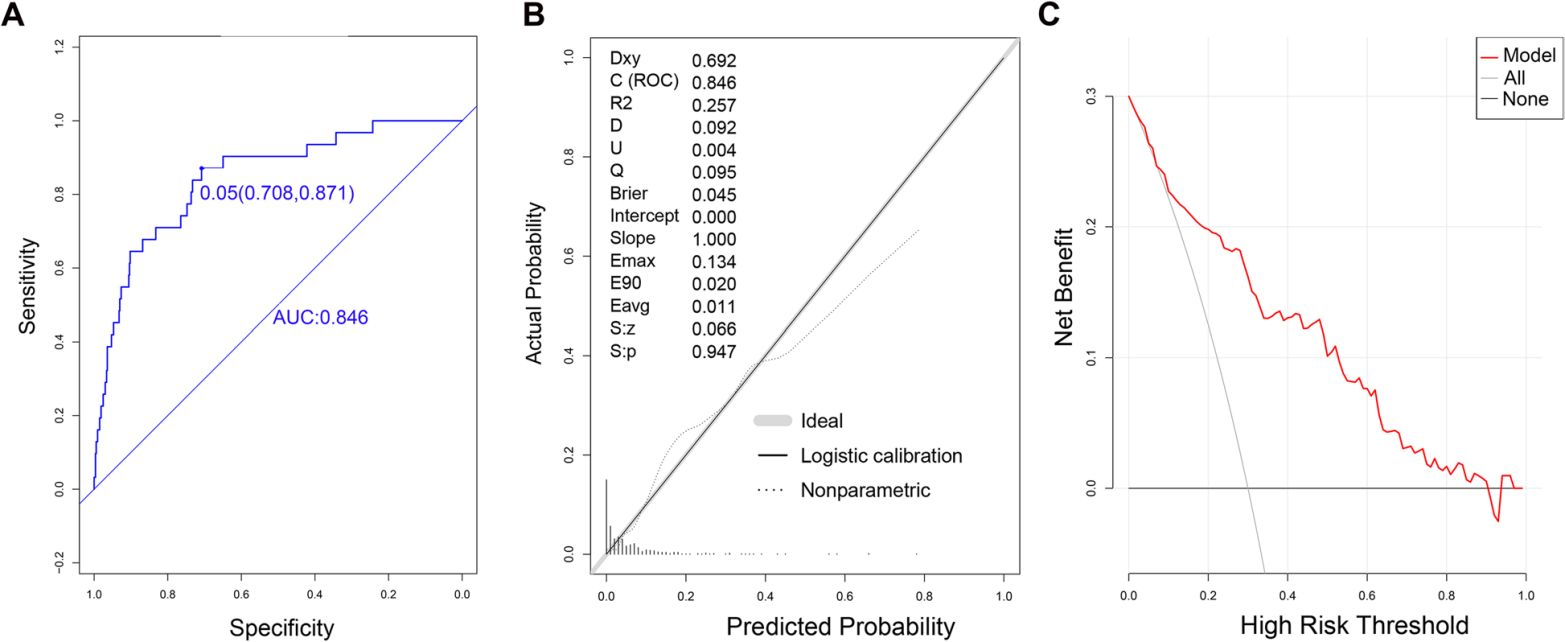
Evaluation of the prediction ability of the established model in the validation group. **A** Discrimination power indicated by ROC curve; **B** Prediction accuracy indicated by calibration chart; **C** Net clinical benefit indicated by DCA

The AUC value for patients in the validation group was 0.846 (95% CI: 0.774–0.918), with an optimal cut-off point of 0.050, a sensibility of 70.8%, and a specificity of 87.1% (Fig. 3A). The prediction accuracy was 0.717 (95% CI:71.6–71.8%), with a PPV of 0.148 (95% CI:9.7–20.0%) and a NPV of 0.989 (95% CI:97.9–99.9%). The *P* value of the calibration chart was 0.947, R2: 0.257, slope: 1.000, Brier: 0.045 (Fig. 3B). Additionally, the DCA curve was far away from the two extreme curves (Fig. 3C).

**Fig. 4:**
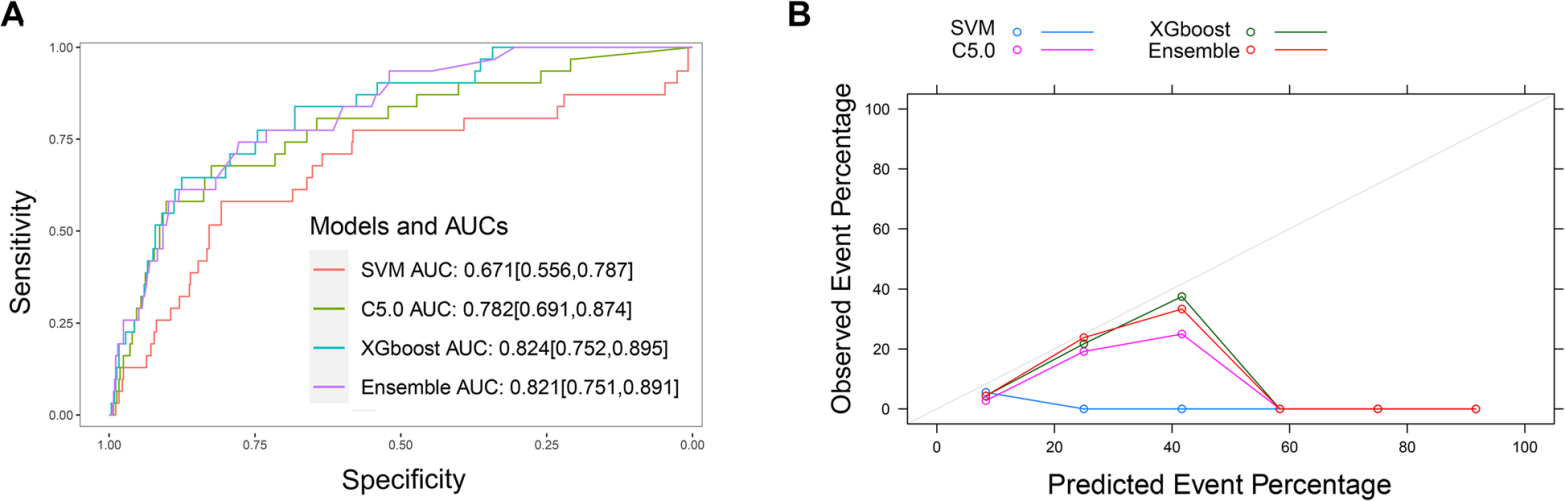
The machine learning models by the ensemble method. **A** ROC curves; **B** Calibration curves

Another model was constructed by the stacking method using SVM, C5.0 and XGboost. The AUC value was 0.821 (Fig. 4A), which was comparable to the logistic model by Delong test (*P* = 0.662). Among the enrolled variables, the top three important variables were the level of pro-BNP higher than 15,000 pg/ml, heart rate and LVEF (Supplementary Fig. 1). However, the calibration of the final integrated model is poor (Fig. 4B).

## Discussion

AMI and its subsequent heart failure are the leading causes of death worldwide [22]. Therefore, early identification of AMI patients with high risks of heart failure during hospitalization could cause doctors’ more attention in the patients’ management. The independent risk factors related to heart failure could be corrected by active inventions, eventually leading to improvement in their survival rate [23, 24]. In this study, the risk factors associated with heart failure in AMI patients during hospitalization were age, heart rate, SBP, LVEF and troponin T and pro-brain natriuretic peptide levels. A logistic model and could efficiently predict the risk of heart failure and the nomogram could visually display how the model predicted the risk.

Previous studies have shown that age is closely related to the incidence of heart failure [12]. Due to slow blood circulation and altered myocardial metabolism in the elderly, the gradual myocardial cell atrophy and insufficient myocardial reserve capacity lead to heart failure after AMI [25]. A number of studies have shown that long-term hypertension eventually leads to heart failure [26, 27]. In fact, hypertension can cause excessive myocardial cell hypertrophy, myocardial cell degeneration and necrosis, eventually leading to heart failure. In addition, patients with heart failure who have lower systolic blood pressure have a worse prognosis than those with higher systolic blood pressure [28]. This phenomenon could be explained by impairment in pump function and cardiac output [28]. In this study, lower blood pressure also contributed significantly to the heart failure in AMI patients. Therefore, for AMI patients with low blood pressure, the blood pressure should be corrected as soon as possible to improve the coronary perfusion pressure and reduce the occurrence of heart failure.

BNP belongs to a family of natriuretic peptides that are mainly secreted by atrial myocytes in the normal heart [29], and its secretion is directly proportional to the severity of left ventricular dysfunction [30]. Brain natriuretic peptide and troponin T have long been used as indicators to predict heart failure [31–36]. Therefore, for patients with high BNP, limiting the blood volume could partly reduce the pressure on the heart by diuresis and other means, finally reducing the incidence of heart failure. This study also showed that heart rate was positively linked to heart failure risk in patients with AMI. This possibly occurs because tachycardia causes cardiac cycle shortening, mainly diastole, and reduces blood return to the heart, resulting in significant reductions in cardiac output per minute and more serious coronary ischemia [37]. Therefore, in the case with left ventricular dysfunction caused by acute myocardial infarction, β-blockers and calcium-channel blockers could be applied to reduce the heart rate, improving the clinical outcome [38]. LVEF is one of the important variables to evaluate the type of heart failure, and was negatively associated with the risk of new-onset heart failure among patients with acute myocardial infarction [17]. This is consistent with the findings of others [39].

In addition, machine learning models by Stacking methods showed no advantage in performance compared with the logistic model. Moreover, the decision support provided by machine learning models is often difficult to interpret [40]. In this study, the prediction model is presented as a nomogram, and has advantages in clinical interpretation and application.

In this study, the established model helped with the early identification of patients at high risk of heart failure. Considering that previous studies mainly focus on the effect of a single factor on the outcomes, this study synthesizes all of the independent factors by establishing a prediction model. This approach makes them intuitive and clearly present, which is helpful for clinician analysis and diagnosis. Compared with the prediction model of Tan et al., our prediction model owned a higher AUC value with less involved factors, indicating a higher discrimination ability [41]. Compared with the prediction model of Yan et al., our model is applicable to a larger population of patients than the elderly women alone [14]. Although studies have shown that women with coronary artery disease have a higher risk of heart failure than men, it is generally accepted that men are more susceptible to cardiovascular disease [39, 42]. Therefore, models that target larger patient populations are highly desirable. The different variables in our prediction model from previous study is due to the variable screening method and patient population heterogenicity. In detail, this study clearly confirmed the relationship between continuous variables and logitp, as well as the absence of collinearity relationship between variables, making it more scientific and accurate compared with previous work. Furthermore, the variables included in our study are more clinically available, which will enable clinicians to quickly identify patients at risk for heart failure [40].

Of course, this study has its limitations. First, case data from only one hospital was selected for this single-center retrospective analysis, and the external validation was not performed due to no data from another center. This limitation might decrease the credibility of the study. Second, data for other important factors, such as socio-economic status and detailed periods before the occurrence of heart failure, were not included due to missing information in the database.

## Conclusion

Age, heart rate, systolic blood pressure, and troponin T, left ventricular ejection fraction and pro-brain natriuretic peptide levels were the independent risk factors for heart failure in patients with AMI. The logistic prediction model can effectively identify patients with high risks of heart failure with good discriminatory power and accuracy. Ultimately, this intuitive and convenient model will be useful for clinicians in AMI patients’ management.

### Supplementary Information

**Additional file 1:** **Supplementary Fig. 1.** The SHAP summary plot of enrolled variables in the machine learning models by the ensemble method

**Additional file 2: Table 1. **The linear relationship analysis between continuous variables and logit (p). **Table 2.** Multicollinear relationship of continuous variables.

## Data Availability

The original contributions presented in the study are included in the article/Supplementary Material, further inquiries can be directed to the corresponding author/s (Email: 13575915795@163.com).
