# Supplementary material for: Establishment and validation of a prediction nomogram for heart failure risk in patients with acute myocardial infarction during hospitalization
Source: BMC Cardiovasc Disord. 2023 Dec 18;23:619. doi: 10.1186/s12872-023-03665-2 (PMC10726532; doi:10.1186/s12872-023-03665-2)
Supplement: Supplementary file 2 — Additional file 2: Table 1. The linear relationship analysis between continuous variables and logit (p). Table 2. Multicollinear relationship of continuous variables. [file 12872_2023_3665_MOESM2_ESM.docx]

## Table1. The linear relationship analysis between continuous variables and logit (p).

| Variables | P |
| --- | --- |
| troponin T | 0.7185 |
| aspartate aminotransferase | 0.7265 |
| white blood cell | 0.7679 |
| age | 0.7266 |
| heart rate | 0.9377 |
| left ventricular ejection fraction, (%) | 0.7507 |

Table2. Multicollinear relationship of continuous variables

| Variables | P |
| --- | --- |
| troponin T | 1.525749 |
| aspartate aminotransferase | 1.501871 |
| age | 1.493079 |
| pro-brain natriuretic peptide |  |
| pro-brain natriuretic peptide500-15000ng/L | 1.380646 |
| pro-brain natriuretic peptide>15000ng/L | 1.612802 |
| white blood cell | 1.238129 |
| C-reactive protein |  |
| C-reactive protein30-80mg/L | 1.199680 |
| C-reactive protein>80mg/L | 1.296713 |
| heart rate | 1.192388 |
| creatine kinase isoenzyme |  |
| creatine kinase isoenzyme25-150ng/ml | 1.173215 |
| creatine kinase isoenzyme>150ng/ml | 1.509055 |
| hematocrit |  |
| hematocrit<0.3L/L | 1.170899 |
| hematocrit>0.45L/L | 1.254869 |
| creatinine |  |
| 100-200μmol/L | 1.135290 |
| 201-400μmol/L | 1.118786 |
| >400μmol/L | 1.163521 |
| systolic blood pressure |  |
| systolic blood pressure>140mmHg | 1.049425 |
| systolic blood pressure<90mmHg | 1.041161 |
| left ventricular ejection fraction, (%) | 1.170398 |
